# The Distribution Characteristics and Ecological Risks of Alkylphenols and the Relationships between Alkylphenols and Different Types of Land Use

**DOI:** 10.3390/toxics11070579

**Published:** 2023-07-03

**Authors:** Yajun Hong, Miao Chen, Ziwei Zhu, Wei Liao, Chenglian Feng, Zhenfei Yan, Yu Qiao, Yaru Mei, Dayong Xu

**Affiliations:** 1 School of Chemical and Environmental Engineering, Anhui Polytechnic University, Wuhu 241000, China; hongyajun@mail.ahpu.edu.cn (Y.H.); xdy826@ahpu.edu.cn (D.X.); 2 State Key Laboratory of Environmental Criteria and Risk Assessment, Chinese Research Academy of Environmental Sciences, Beijing 100012, China; chenmiao@tongji.edu.cn (M.C.); 210205020012@hhu.edu.cn (Z.Y.); qiaoyu202@mails.ucas.ac.cn (Y.Q.); 3 Wetland Research Center, Jiangxi Academy of Forestry, Nanchang 330032, China; ziweiangel@163.com (Z.Z.); collect21@sina.com (Y.M.); 4 College of Environment, Hohai University, Nanjing 210098, China

**Keywords:** emerging contaminants, endocrine disrupting chemicals, alkylphenols, distribution characteristics, ecological risks, types of land use

## Abstract

In this study, the spatial distribution characteristics of nine alkylphenols (APs) in the Yongding River and Beiyun River were analyzed. The differences in the concentrations and spatial distribution patterns of nine APs were systematically evaluated using principal component analysis (PCA). The relationships between the concentration distribution patterns and the risks associated with nine APs were investigated under various categories of land use conditions in the region. The results demonstrated that the APs were widely present in both rivers, and the pollution risks associated with the APs were more severe in the Yongding River than in the Beiyun River. The results show that the contamination risks associated with 4-NP were the most serious in the two rivers, with detection percentages of 100% and 96.3%, respectively. In the Yongding River, the APs showed a tendency of low concentration levels in the upper reaches and high levels in the middle and lower regions. Meanwhile, the overall concentration levels of the APs in the Beiyun River were relatively high. However, despite the differences between the upper and middle regions of the Yongding River, the distribution pattern of the APs in the Beiyun River was basically stable. The concentration levels and risk quotient of the APs were negatively correlated with the vegetation cover land use type and positively correlated with the cropland and unused land use types within 500 m, 1 km, and 2 km. The purpose of this study was to provide theoretical data support and a basis for AP pollution risk evaluations in the Yongding River and Beiyun River.

## 1. Introduction

The rivers of the world provide important ecosystems for many forms of life, as well as valuable goods and services to humanity [1]. Regional land use analysis is the main source of information for assessing the extent to which social, economic, and environmental factors influence urbanization processes and spatial structures [2]. Changes in land usage and land coverage will impact the structures and functions of ecosystems and are important driving factors of the changes in ecosystem services. The research regarding such change processes plays a decisive role in maintaining ecosystem services [3,4]. Urbanization levels have been unceasingly increased with the rapid development of social economies, resulting in surges in population. The emissions of industrial wastewater and sewage into nearby water resources have been steadily increasing. Unfortunately, environmental infrastructure construction has lagged, resulting in large amounts of untreated wastewater entering rivers. River water pollution has become a serious threat to the stability of water ecological systems [5,6]. One of the most important rivers in China is the Yongding River, also known as the “mother river”. The Beijing-Tianjin-Hebei Region is an important headwater area containing many ecological barriers and corridors [7,8,9]. However, the healthy development of the economy and society of the Beijing-Tianjin-Hebei Region has been severely restricted by such outstanding problems as damage to the ecosystem, excessive water resource development, and the environmental bearing capacity [10]. The Beiyun River is the largest river system in the Beijing Plain Basin. The Beiyun River is mainly replenished by unconventional water sources, such as effluent from the municipal sewage treatment plant. The sewage discharge volume of the entire river basin is approximately 3 million m^3^/d, making it the most seriously polluted river among the five major river systems in Beijing, with high risk of pathogenic microorganisms [11]. Therefore, it is of major significance to strengthen the management and governance of the rivers to improve water quality and fully enhance the utilization rates of the water resources. Such improvements will have beneficial impacts on the ecosystems and the maintenance of ecological stability, as well as protecting the health of the population and increasing life satisfaction.

Alkylphenols (APs) are typical endocrine-disrupting chemicals (EDCs). APs are extensively used as feedstock for alkylphenol polyoxyethylene ethers (APEOs). They are used in the production of phenolic resins, heat stabilizers, antioxidants, and hardeners [12,13,14]. APs in a water environment are mainly produced by the biodegradation of long-chain ethoxylates. Those ethoxylates can be removed by conventional wastewater treatment technologies [15,16], but APs may be more persistent, lipophilic, and toxic than their precursors [14]. They tend to be present in higher concentrations in the environment compared with other EDCs [17,18]. APs have been continuously detected in river water and sediment, usually at the concentration levels of ng/L or ng/g. The results of the study conducted by Lei et al. [19] confirmed that nonylphenol (NP), octylphenol (OP), and bisphenol A (BPA) could be detected in 100% of the urban rivers in Beijing, Tianjin, and Hebei, with concentration levels between 23 and 255 ng/L. Cheng et al. [20] showed that the concentrations of NP, OP, and BPA in the Yongjiang River Basin were 140–3948, 6–828, and 15–1415 ng/L, respectively. Li et al. [21] determined that the concentrations of three phenolics (NP, OP, and BPA) in the Pearl River sediment varied from 204.4 to 12,604.3, 32.6 to 297.3, and 12.8 to 298.4 ng/g, respectively. In the river sediment of the Duliujian River, the concentration levels of those same substances ranged from 153.5 to 3614.9 ng/g, 90.7 to 990.0 ng/g, and 83.5 to 913.3 ng/g, respectively. Although the above-mentioned concentration levels of APs will not cause serious acute toxicity to aquatic organisms, they will potentially damage their endocrine systems, which has become especially evident in fish sampled from the region [21,22] APs can enter the body in many ways, including through diet and respiration, and they can cause diseases in the human reproductive, cardiovascular, and immune systems [23].

However, the previous research in the Yongding River and Beiyun River has focused on the degrees of heavy metal pollution in the water bodies and the sediment of the rivers [24]. In addition, the water ecological carrying capacities, river restorations, and evaluations [8]; persistent organic pollutants and microplastics [9]; distributions of plankton and microorganisms [11]; and the monitoring of water quality indicators have all been major concerns. To effectively protect the environment and human health, it is also important to determine the pollution levels and characteristics of the distribution patterns of APs in the water environment of the Yongding River and Beiyun River. In this study, 35 water samples were obtained from the Yongding River and Beiyun River (Beijing section). The concentration characteristics of the APs in the river water were analyzed, and the relationships between the concentration distribution pattern and risk potential of the APs and the various land use types in the region were investigated. The aim was to provide a scientific foundation for the study and management of pollution and improve the water quality, as well as protecting aquatic life in the Yongding River and Beiyun River.

## 2. Materials and Methods

### 2.1. Chemicals and Reagents

The basic chemical information data are detailed in Table S1. The 4-EP, 2-PPP, 4-t-BP, 2-n-BP, 4-PTP, 4-HXP, 4-HTP, 4-OP, 4-NP, BPA, 4-t-OP, 4-n-NP, and the internal standards (4-EP-d_4_, 4-t-OP-^13^C_6_, and 4-n-NP-d_4_) were obtained from TRC (Toronto, ON, Canada). The HPLC-grade methanol (MeOH), acetonitrile (ACN), and ethyl acetate (ACETATE) were acquired from Fisher Scientific (USA). The hydroxide (NH_4_OH, 14 M) used in this study was supplied by Sigma Aldrich (USA). The ultra-pure water (>18.2 MΩ/cm) was obtained using the Milli-Q Advantage Purification System (Millipore, USA). Stock solutions of each compound and the internal standards were taken at a concentration of 1000 mg/L in MeOH and stored in amber brown bottles at −18 °C under dark conditions before use. The working standard solutions (10 mg/L) were obtained by serial dilution prepared from the stock solution and renewed monthly to eliminate destabilizing effects. In addition, calibration standards with gradient concentrations (0, 5, 10, 50, and 100 μg/L) of analytes and 50 μg/L internal standard solutions were also prepared.

### 2.2. Sample Collection and Preparation

**Figure 1:**
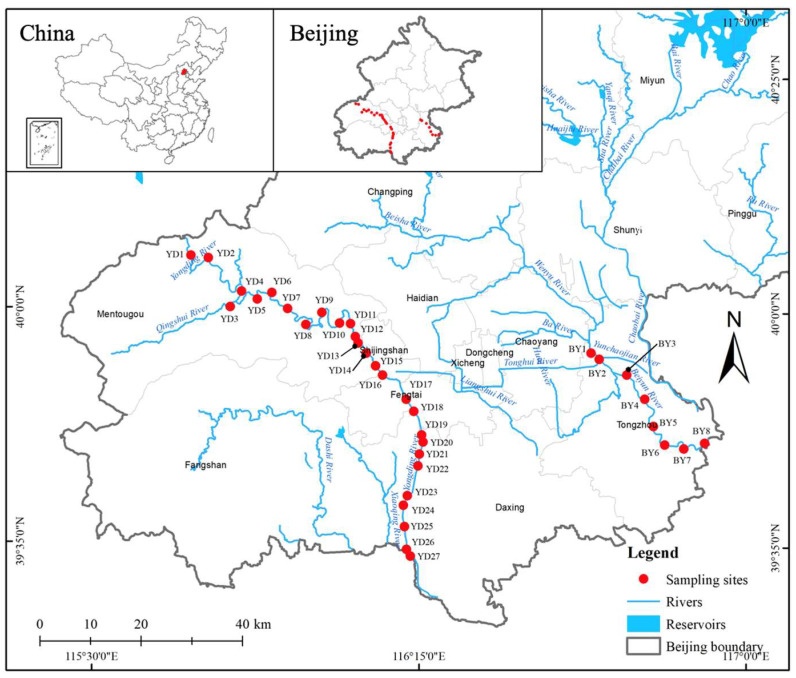
Sampling locations in the Yongding River and the Beiyun River.

The study targets were the Yongding River and the Beiyun River (Figure 1). Thirty-five samples (27 from the Yongding River and 8 from the Beiyun River) were collected in January of 2022. Pretreatment of the collected samples was performed according to previous methods [21,25,26].

### 2.3. Sample Analysis

Ultra-Performance Liquid Chromatography (UPLC) separation was performed using a Waters ACQUITY UPLC device (Waters, USA). The instrumental conditions were as follows. The column temperature was set at 40 °C, and the sampling volume was 2 μL. MQ water with 0.01% NH_4_OH (Solvent A) and MeOH (Solvent B) were used as the flowing phase, with a flow rate of 0.2 mL/min. All of the analytes were identified according to their retention times and targeted ion pairs as per the standards. The optimized parameters of the mass spectrometry for the analytes are listed in Table S1. Quantification of these chemicals was performed using the internal standard method.

### 2.4. Quality Assurance and Control Measures

It was found that the calibration curves for the selected chemicals showed strong uniformity over a broad range of levels (R^2^ > 0.99). As shown in Table S1, the method detection limit (MDL) and method quantification limit (MQL) for the surface water samples ranged from 0.05 to 0.15 ng/L and 0.2 to 1.8 ng/L, respectively. The results of the study showed that the sample recoveries of the target analytical analytes in the two rivers were from 74% to 88%, 72% to 78%, and 71% to 87% at the water-spiked levels of 10, 50, and 100 ng/L, respectively. The instrumental quantification limit (IQL) was 10 times that of the signal-to-noise (S/N) ratios, and the MDL and MQL were 3 and 10 times that of the S/N ratios, respectively.

### 2.5. Principal Component and Difference Analyses

The relationships between the distribution characteristics of the APs in the two rivers were explored in this study. Reductions were made to the data dimensions since correlations may have existed between multiple variables, which increased the difficulty and complexity of the analysis process. This resulted in moderate reductions in the number of indicators to be analyzed. The possibility of loss of information included in the original metrics was minimized to achieve a well-rounded analysis of the gathered data [27]. Principal component analysis (PCA) was used to map the N-dimensional characteristics to k (2–3) dimensions. The obtained feature represented a new orthogonal feature, also called the principal component, which was a reconstruction of the k-dimensional characteristics anchored on the original N-dimensional characteristics [27]. In this study, PCA was used to fit the corresponding functional relationships between the content of nine AP species and the main ranking axis. The results showed the spatial allocation characteristics of the APs in the two examined rivers. PCA dimensionality reduction analysis was completed using Canoco 5 software, and the sample grouping results were verified by ANOVA in R software.

### 2.6. Spatial Analysis

The geospatial parameters, such as the spatial distances to the outlets and the topological distances between all sites, were obtained using the network analysis tool in ArcGIS software version 10.2 [28]. The digital height model data (250 m resolution) obtained from the Shuttle Radar Topography Mission (SRTM) V4.1 dataset were used to define the watershed basin boundaries and watercourse features with the hydrology tool in ArcGIS software [29]. The category system in CNLUCC (http://www.resdc.cn/data.aspx?DATAID=264, accessed on 11 January 2023) was also referenced in this study. The patterns of the land usage were classified into six first-class types as follows: cropland (paddy fields and dry land areas); forested land; grassland; water areas (rivers, pools, and reservoirs); impervious areas (residential, industrial, and mining cover areas); and unutilized land (desert, marshland, and bare land areas) [28]. Three buffer regions were the focus within a 500 m, 1 km, and 2 km radius, respectively, for the upstream area of each site. The percentages for the six land use types were calculated, and they are detailed in Tables S2 and S3. The range used in this study describes a contiguous continuum of human activity from the local to the regional scale. The buffering tool in ArcGIS software was utilized to extract the land use parameters within the aforementioned buffers [28]

## 3. Results and Discussion

### 3.1. Distribution Characteristics of the Concentration Levels of the APs

The detected concentration and frequency values of the APs in the two examined rivers are summarized in Table 1. As can be seen from the table, 4-HXP was not detected at all points in the Yongding River. However, the other eight APs were all detected, among which 4-NP and 4-OP had the highest detection rates at 100% and 88.9%, respectively. The detection rates of 2-PPP and 4-t-BP were over 70%. It was also found that 2-PPP, 4-t-BP, and 4-PTP were detected at all the sampling points, but 4-EP, 4-HXP, and 4-HTP were not. The detection rate of 4-NP was as high as 96.3%, indicating that AP pollution was widely present in the Yongding River and Beiyun River. The concentration levels of the APs were at the ng/L level. AP pollution risks in the Yongding River were more serious than those in the Beiyun River, with the 4-NP pollution found to be the most severe.

The detection rates of 4-NP were high in both examined rivers, which was consistent with the results of earlier studies [19]. This was determined to be due to the presence of synthesized nonylphenol polyoxyethylene ether (NPEO), the world’s second most abundantly used non-ionic surface-active agent. NPEO is widely used in the pulp and paper making, textile manufacturing, agriculture, metal and plastic manufacturing, and oil refining industries. Products containing NP include detergents, emulsifiers, wetting and dispersing agents, antistatic and emulsifying agents, and solubilizers, which are used in a wide range of industrial, institutional, commercial, and domestic applications [30]. NPEO, as an important component of the product, enters water bodies in various ways. It is easily degraded to NP, with a more stable chemical structure under the combined actions of various environmental factors. Due to recent rapid urbanization, modernization, and industrialization, large amounts of NP have entered rivers, lakes, and reservoirs. It is estimated that approximately 60% of the NP (and its derivatives) produced in the world has been introduced into water resources [22]. Theoretically speaking, there are more than 100 types of NP structural isomers, of which 4-NP accounts for approximately 90% of them. Sewage treatment plants are unable to effectively degrade parts of NP using traditional methods [22]. This study observed that there were many urban residential areas, hospitals, factories, sewage plants, etc. located along the banks of the Yongding River and Beiyun River. The high concentration levels and detection rates of NP in the rivers were related to the large quantities of nondegraded NP in the discharged sewage and wastewater.

### 3.2. Spatial Distribution of the Concentrations of APs

**Figure 2:**
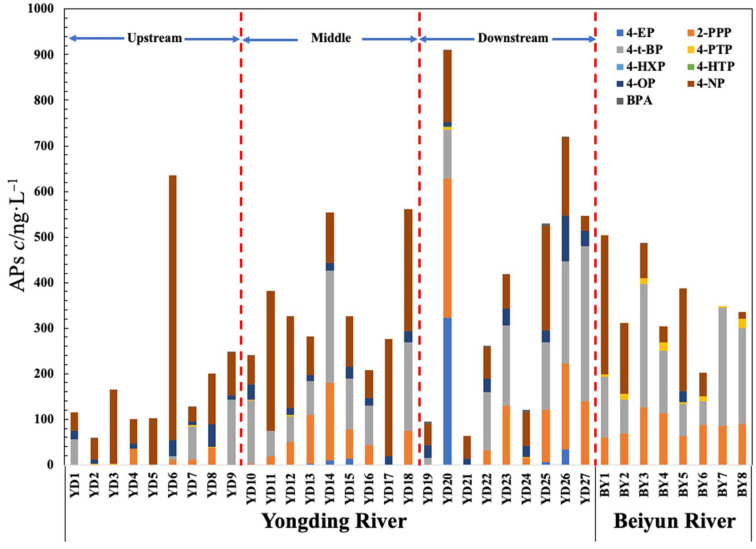
Concentration distribution patterns of the APs at various sampling points in the Yongding River and Beiyun River. Note: In the figure, the concentration levels of each of the nine APs are shown in different colors. Column heights of the stacking plots indicate the total concentration of all APs at those points.

In this study, the Yongding River was divided into upstream (Points YD1–YD9), middle stream (Points YD10–YD18), and downstream (Points YD19–YD27) sections. The concentration distribution patterns of the APs at each sampling point along the Yongding River and Beiyun River are shown in Figure 2. The concentration distribution of the APs in the Yongding River generally presented a trend of lower concentrations in the upstream region and higher concentrations in the middle and lower regions. The middle section of the Yongding River was found to have high concentration values of APs, with a total concentration above 200 ng/L. This was attributed to the middle section passing through the Mentougou District, Shijingshan District, and Fengtai District, which were characterized by high population densities and a variety of residential communities, hotels, hospitals, sewage plants, and so on. The relatively high concentration levels of APs at the middle section’s sampling points were due to the abundant use of APs in the production and living activities of those areas. Point YD20 in the downstream section was determined to be the point with the highest total concentration of APs, possibly due to the location of the tributary of the Yongding River: the Yongding River Main Canal. At that particular sampling point, the river passed through many residential areas and received large amounts of sewage discharge, with very poor water quality and a certain smell detected.

The downstream sampling points revealed that the AP concentrations were lower in those areas. The majority of the points were located in swamps or wetland parks (such as Points YD19 and YD21) or in forested parks and green dam areas upstream (such as Century Park), which were relatively free of pollutants due to water purification and adsorption. In addition, Point YD24 in the upstream section was in an area where the river flowed through various golf clubs and country parks, with lower population densities and scenic environmental conditions, resulting in relatively low concentration levels of APs in the water samples. However, the downstream section also passed through densely populated villages with many agricultural activities, leading to increases in the concentration levels of the APs in those sections. The sampling results also revealed several points along the Beiyun River where the total concentration of APs was relatively high. This was related to the Beiyun River’s water distribution in the Beijing Tongzhou District deputy center downstream plains, which accommodates 90% of the central city drainage task [31]. The many tributaries which participate in the Beiyun River’s drainage of rain and sewage may have also had influencing effects on the sampling results. Since the drainage water mainly comes from recycled water, the river has both typical and unconventional water sources. The river sediment becomes silted with the discharge of pollutants, resulting in high levels of AP pollution [32]. The pollutants in the river sediment are then rereleased into the water bodies over time.

### 3.3. Spatial Distribution Differences in the APs

**Figure 3:**
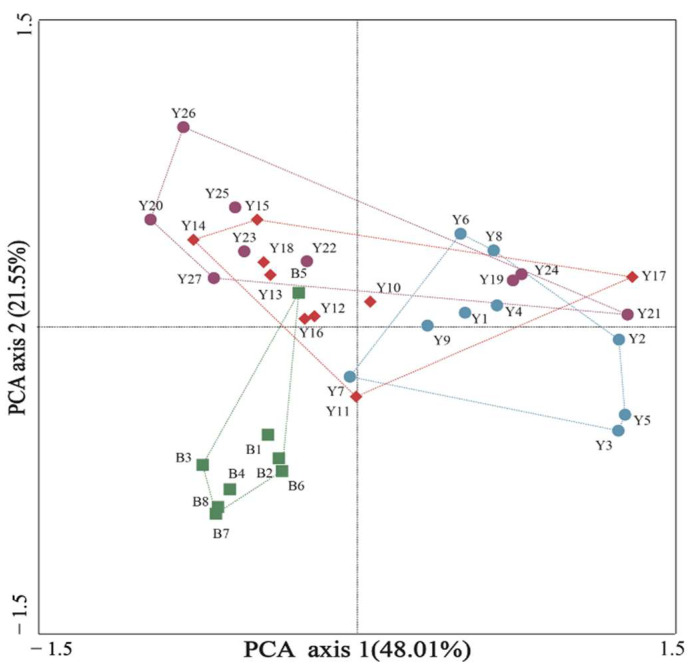
Principal component analysis diagram of the sampling points in the Yongding River and the Beiyun River.

PCA was performed on the distribution of all APs at 27 points along the Yongding River and 8 points along the Beiyun River in order to have a better comprehension of the differences in the spatial distribution patterns. The results are shown in Figure 3. The PCA and regional significance analysis focused on nine sampling sites in the upper reaches of the Yongding River (Points YD1–YD9); the middle section of the Yongding River (Points YD10–YD18); the lower reaches of the Yongding River (Points YD19–YD27); and the Beiyun River, and significant differences were observed (ANOVA, R = 0.186, *p* = 0.005 < 0.05). The sampling sites of the Yongding River were roughly divided into three sections: upper, middle, and lower sections. The upper reaches of the sampling points were basically assessed together, as were the downstream sampling points. It was confirmed that the levels of AP concentrations in the middle and downstream segments were higher than those in the upstream segments. However, there were also some abnormal sampling results observed. With the exception of Point BY5, all the sampling points of the Beiyun River were clustered together, which verified that the water sources were mainly unconventional (wastewater treatment plant return water, agricultural irrigation return water, etc.), and the pollutant concentration levels were basically stable throughout the Beiyun River [32].

**Figure 4:**
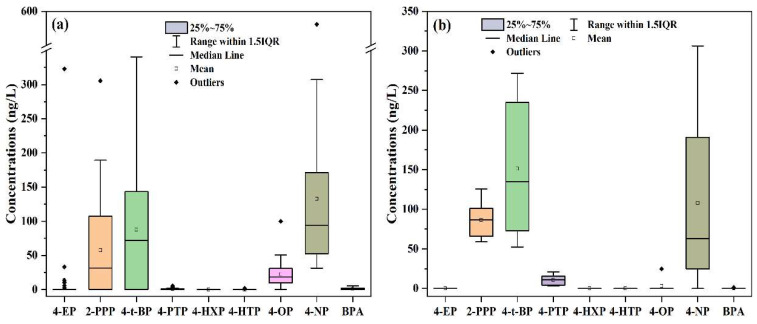
Analysis of the distribution differences of the AP concentrations at different sampling points in the (**a**) Yongding River and (**b**) Beiyun River.

The differences in the distribution patterns of the AP concentrations in the Yongding River and Beiyun River sampling sites were further analyzed to examine the distribution differences in the AP concentration levels at the different sampling sites, as detailed in Figure 4. Sampling Points YD20 and YD26 were in the lower reaches of the Yongding River, with many residential areas, hospitals, and sewage plants nearby. The generated sewage discharge was considered to be one of the important sources of the high concentrations of APs [33]. However, Sampling Point YD6 was located in the upper reaches of the Yongding River, with no serious sources of pollution nearby. As can be seen in Figure 2 and Figure 4b, the sampling results revealed that 4-OP, 4-NP, and BPA displayed high values at Sampling Points BY5, BY1, and BY3 in the Beiyun River. Sampling Point BY1 was located in the upper reaches of the Beiyun River and Wenyu River, with many schools, residential communities, and hospitals nearby. Sewage containing 4-NP may have been discharged into the river at that location, resulting in the observed higher AP concentration values in that section.

As can be seen in Figure 3, the PCA and sampling site difference analysis results indicated that detection differences existed between the upper and lower reaches of the Yongding River. The high urbanization rates and high proportions of industrial and domestic land in the middle and lower reaches of the Yongding River resulted in higher AP values, while the upper reaches were in mountainous areas with less pollution. However, the distribution patterns of the APs in the Beiyun River remained generally stable. The research results obtained by Yu et al. [34] showed that there are certain correlations between the water quality and types of land use. The effects of new pollutants with different spatial distributions were observed in other research conducted in the area, and the types of land use were determined to be the main influencing factors [35,36]. However, it was considered that in addition to the types of land use, the distribution patterns of the APs and other new pollutants could also be related to the plant cover density, population density, proportion of farmland, and the economic living standards in different regions [37]. Therefore, this study further analyzed the influencing effects and weight correlations of each factor based on relevant data.

### 3.4. Relationships between the Types of Land Use and the Concentrations and Risk Quotient of the APs

**Figure 5:**
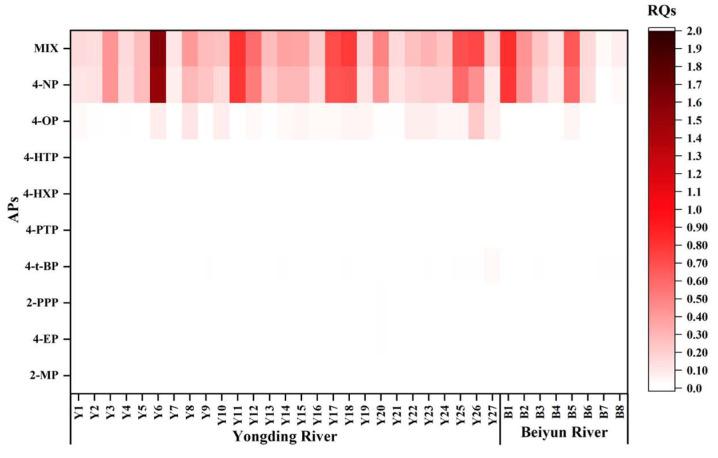
Heat plot of the ecotoxicological risks (represented by chronic RQs) of each AP (*y* axis) for aquatic organisms in the Yongding River and Beiyun River sampling sites (*x* axis).

**Figure 6:**
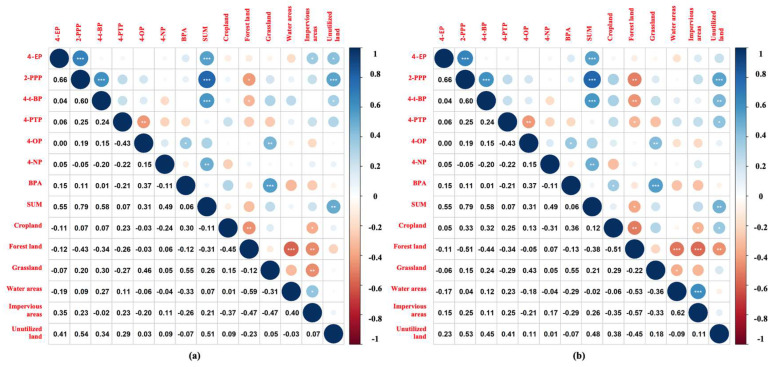

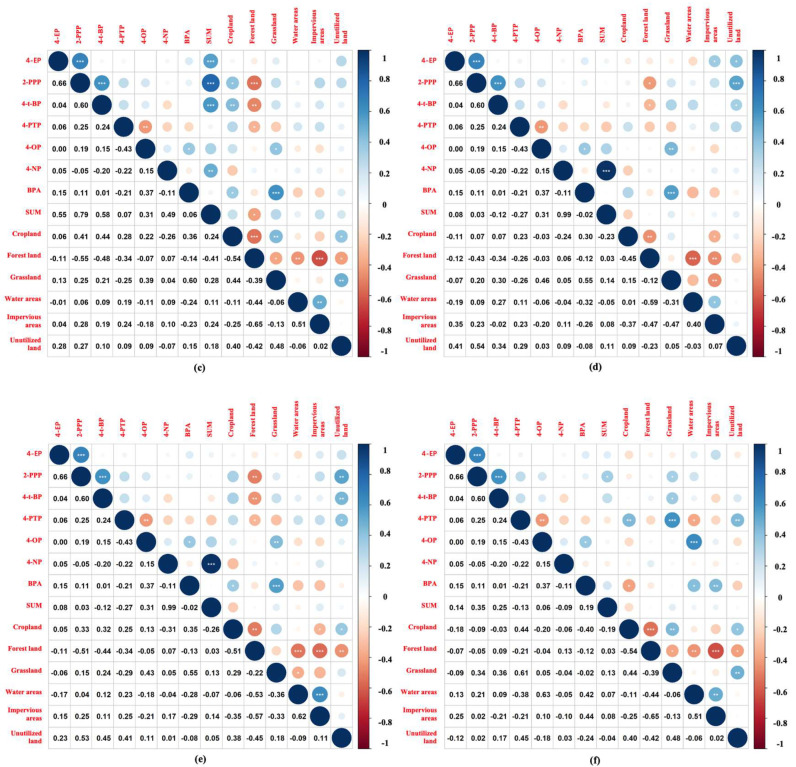
Relationships between the land use patterns and the concentration levels and risk quotient of the APs. (**a**–**c**) Relationships between the land use modes and concentration distributions of the APs within 500 m, 1 km, and 2 km, respectively. (**d**–**f**) Relationships between the land use modes and the risk quotient of the APs within 500 m, 1 km, and 2 km, respectively. Note: In the figure, the blue circles indicate positive correlations, and red circles indicate negative correlations. The larger the circle, the greater the correlation. * *p* < 0.05. ** *p* < 0.01. *** *p* < 0.001.

The extent to which land use affects the ecological risks of pollutants may vary from region to region. Therefore, the relationships between the potential risks of new pollutants, as well as the management of buffer landscapes and land use scales in different watersheds, require further consideration. This process can provide a scientific basis for maintaining or improving living standards and formulating land management policies. In our previous studies [26], the ecological risks of AP pollution in the Yongding River and Beiyun River were reported in detail (Figure 5). In this investigation, three buffer zones at each site (500 m, 1 km, and 2 km) were the focus in the upstream areas, and the percentages of the six main land use types (cropland, forested land, grassland, water areas, impervious areas, and unutilized land) were calculated, as detailed in Tables S2 and S3. The relationships between the land use types and the concentration levels and risk quotient of the APs were examined, as shown in Figure 6.

Figure 6 shows that the concentration levels and risk quotient of the APs were negatively correlated with the forested land use type within 500 m, 1 km, and 2 km, which indicates that forested land use may reduce the concentrations and risk quotient of APs. Moreover, the concentration levels and risk quotient of the APs were found to be positively correlated with the cropland and unutilized land use types within 500 m, 1 km, and 2 km, indicating that the cropland areas were important sources of APs in the water. Other sources of APs were not represented, such as industrial, medical, and other land use types, and may have been unutilized land.

Land use types are major factors influencing the exposure and distribution of new contaminants. Land use types associated with human activities, such as croplands and impervious surfaces, may increase pollutant concentrations. On a global scale, the intensification and expansion of anthropogenic land use types are the most important drivers of water quality degradation [38,39]. The higher the proportion of urban areas among the land use types, the higher the potential for pollution by APs [40]. It has been determined that agricultural land and urban sewage are the largest sources of diffuse pollution in freshwater systems [41]. In addition, pollutant concentrations are significantly and negatively correlated with vegetation cover, such as forests and grasslands [40]. On one hand, forests and grasslands promote the uptake of pollutants and play important roles in improving water quality and reducing pollution by APs [41,42]. On the other hand, vegetation cover provides a buffer zone which slows down the diffusion of pollutants to some extent.

In general, agricultural land and built-up areas are major sources of nutrients and pollutants in surface water, which increases their chemical risks to various species [43]. As the proportion of cropland increases, biodiversity decreases, and habitats change [44]. In addition, runoff increases as urban or agricultural land cover expands and decreases as vegetated land increases, and the loss of forested areas increases erosion and sedimentation, alters water flow and thermal conditions, and affects carbon and nutrient cycling, which may impact the chemical risks in aquatic ecosystems [45].

## 4. Conclusions

This study’s research results confirmed that alkylphenols (APs) are widely present in the Yongding River and Beiyun River. The concentration levels of AP pollution in the Yongding River were determined to be more serious than those in the Beiyun River. Among the nine examined APs, 4-NP pollution was the most severe in the two rivers, with detection rates of 100% and 96.3%, respectively. Based on this study’s findings, it will be necessary to examine effective measures for the reduction of AP pollution, especially 4-NP pollution, in order to promote the normal growth and reproduction of aquatic organisms, avoid damaging the normal structures and functions of aquatic ecosystems, and ensure the sustainability of the future use of river water resources.

## Figures and Tables

**Table 1:** Concentration and frequency values of the APs detected in the Yongding River and Beiyun River (ng/L).

APs	Yongding River					Beiyun River				
	MAX	MIN	MED	AVE	Detected No.	MAX	MIN	MED	AVE	Detected No.
4-EP	322.9	ND	ND	14.4	6	ND	ND	ND	ND	0
2-PPP	305.4	ND	31.6	57.6	19	125.5	59.0	86.7	86.5	8
4-t-BP	340.6	ND	71.7	88.0	19	271.6	52.4	135.2	151.2	8
4-PTP	5.6	ND	ND	0.8	9	21.1	3.2	11.2	10.8	8
4-HXP	ND	ND	ND	ND	0	ND	ND	ND	ND	0
4-HTP	1.7	ND	ND	0.1	1	ND	ND	ND	ND	0
4-OP	99.9	ND	18.4	22.5	24	24.8	ND	ND	3.1	1
4-NP	580.8	31.4	93.7	133.0	27	306.3	ND	63.1	108.0	7
BPA	5.3	ND	ND	1.2	11	1.3	ND	ND	0.2	1

## Data Availability

The data presented in this study are available on request from the corresponding author upon reasonable request.

## Supplementary Materials

The following supporting information can be downloaded at https://www.mdpi.com/article/10.3390/toxics11070579/s1: Table S1: Basic information of target analytes and parameters for mass spectrometry optimization. Table S2: The relationship between six land-use types and concentration at three buffer regions interpreted from Landsat 8 remote-sensing images. Table S3: The relationship between six land-use types and risk quotient at three buffer regions interpreted from Landsat 8 remote-sensing images.
